# Epigenetic memory and allostatic integrity: a theoretical integration of the hierarchical-circular model using a longitudinal methylation signature of dementia

**DOI:** 10.3389/fnagi.2026.1919708

**Published:** 2026-09-22

**Authors:** Samuel Ruesga Mundo

**Affiliations:** Department of Psychiatry, Regional General Hospital No. 180,Instituto Mexicano del Seguro Social (IMSS), Tlajomulco de Zúñiga, Jalisco, Mexico

**Keywords:** allostatic integrity, allostatic load, biological memory, dementia, DNA methylation, epigenetic memory, hierarchical-circular model, PNEI network

## Abstract

**Introduction:**

The Hierarchical-Circular Model of Biological Memory (HCM) proposes that dementia results from disrupted circular information flow across five levels: genetic, epigenetic, allostatic, PNEI network, and interoceptive. Allostatic integrity—the adaptive capacity for circular flow—determines whether pathology translates into dementia.

**Objective:**

To evaluate whether a recently published blood DNA methylation signature for incident dementia is consistent with the predictions of the HCM.

**Methods:**

We systematically mapped Zhang et al.’s findings (44 CpGs, 44 DMRs, pathway enrichments, methylation risk score) to HCM levels and predictions. We interpreted these findings as providing indirect and conceptual support for the model rather than direct empirical validation.

**Results:**

The 44 CpGs/DMRs linked to dementia were enriched in immune and metabolic pathways. Their methylation risk score predicted cognitive decline independently of age, sex, APOEε4, education, and MMSE. These findings are consistent with the HCM’s Level 2 (epigenetic memory), Levels 3–4 (allostatic load/PNEI), and Predictions 1 (composite biomarkers outperform single) and 4 (allostatic integrity moderates reserve).

**Discussion:**

Epigenetic changes precede dementia, supporting Level 2 as an early event. Immune/metabolic pathways link to allostatic load. Independence from APOEε4/education is consistent with allostatic integrity being distinct from genetic risk and reserve.

**Conclusion:**

Zhang et al.’s study provides large-scale evidence that blood DNA methylation may serve as a peripheral biomarker of lost allostatic integrity, consistent with the HCM and supporting a shift from linear to circular, multilevel frameworks for dementia.

## 1 Introduction

Alzheimer’s disease and related dementias (ADRD) represent one of the greatest public health challenges of the 21st century, with global prevalence expected to rise sharply (GBD 2019 Dementia Forecasting Collaborators, 2022). Despite decades of research and unprecedented advances in neuroimaging, biomarkers, and genetics, disease-modifying therapies have produced only modest clinical benefits, and most pharmacological trials continue to fail (Cummings et al., 2024). This therapeutic impasse has fueled growing recognition that predominant linear models—centered on beta-amyloid, tau, and single-pathway cascades—are insufficient to capture the full complexity of dementia pathogenesis (Long and Holtzman, 2019; Serrano-Pozo et al., 2024).

Recent conceptual and diagnostic advances have deepened, rather than resolved, this paradox. The 2024 NIA-AA biological criteria formally define Alzheimer’s disease by its underlying pathology and biomarker profile, yet a persistent dissociation remains between neuropathological burden and clinical expression: individuals with substantial amyloid and tau pathology may remain cognitively intact, whereas others with relatively modest biomarker changes exhibit rapid decline (Savva et al., 2009; Jack et al., 2024). These discrepancies suggest that dementia cannot be fully understood as a simple downstream consequence of aggregated proteins, but instead reflects broader disturbances in how biological systems encode, integrate, and stabilize information over time.

Converging evidence from large longitudinal cohorts has highlighted the importance of lifespan, multisystem influences. Elevated allostatic load—a composite index of cumulative physiological “wear and tear” across metabolic, cardiovascular, and inflammatory systems—is prospectively associated with increased risk for all-cause dementia and specific subtypes, including Alzheimer’s disease (Ruan et al., 2025; Palix et al., 2025; Souza-Talarico et al., 2025). Early-life stress, childhood adversity, and socioeconomic hardship across the life course have also been linked to higher dementia risk (Livingston et al., 2020; Lee et al., 2025), underscoring the role of social and psychological stressors in shaping long-term vulnerability.

At the molecular level, gene–environment interactions and epigenetic mechanisms provide a plausible route by which early and chronic stress exposures become biologically embedded. Work on stress-responsive genes such as FKBP5 and NR3C1 has demonstrated that specific genetic variants modulate epigenetic plasticity, HPA axis reactivity, and neurophysiological activity patterns across the lifespan (Klengel et al., 2013; Zannas et al., 2017; Yusupov et al., 2024; Nöthling et al., 2025). These data reinforce the idea that genetic architecture and epigenetic “memory” jointly calibrate stress responses and neural network function, thereby influencing who will convert from mild cognitive impairment to dementia under comparable neuropathological conditions (Panitch et al., 2024).

In parallel, advances in systems neuroscience and interoception research suggest that dementias involve fundamental disruptions in how the brain tracks, predicts, and regulates bodily signals (Critchley and Garfinkel, 2017; Khalsa et al., 2018; Hazelton et al., 2025a, 2025b). Disturbances in interoceptive accuracy and network connectivity have been implicated in behavioral-variant frontotemporal dementia and Alzheimer’s disease, suggesting that disruptions in the dynamic integration of bodily and environmental information may constitute a transdiagnostic mechanism of cognitive and behavioral decline.

Taken together, these strands of evidence reveal a fragmented but convergent picture: dementia risk and progression are shaped by interactions among genetic predisposition, epigenetic molecular memory, cumulative allostatic load, multisystem (PNEI) dynamics, and interoceptive-neuronal integration (McEwen and Stellar, 1993; Danese and McEwen, 2012; Slavich and Irwin, 2014; Feng et al., 2025). However, current models typically examine these components in isolation, lacking a unifying framework capable of explaining how perturbations at one level propagate across others to produce stable, maladaptive states (Long and Holtzman, 2019).

### 1.1 The hierarchical-circular model of biological memory

The Hierarchical-Circular Model of Biological Memory (Ruesga Mundo, 2026a,b,c) proposes a fundamental reconceptualization of Alzheimer’s disease and related dementias. Rather than viewing dementias as linear consequences of aggregated proteins, the model frames them as emergent outcomes of disrupted circular information flow across five interconnected levels:

1. Morphogenetic programming and genetic architecture – The genetic blueprint, including APOE variants and polygenic risk, that establishes baseline vulnerabilities and resilience capacities.
2. Epigenetic molecular memory – DNA methylation, histone modifications, and non-coding RNA regulation that encode environmental signals and shape future cellular responses.
3. Allostatic load and systemic physiological adaptation – The cumulative physiological consequences of repeated or chronic adaptation to environmental demands, measured through multisystem biomarkers.
4. Psycho-Neuro-Endocrine-Immunological (PNEI) network – The bidirectional communication pathways linking psychological states, neurological function, endocrine signaling, and immune activity.
5. Interoceptive-neuronal integration – The integration of sensory information about the internal state of the body with neuronal dynamics across large-scale brain networks.

The model is organized around the unifying principle: Signal → Plasticity → Stable State. At any level, perturbation can propagate bidirectionally through the system, establishing maladaptive stable states that manifest clinically as dementia. The concept of allostatic integrity—the capacity of the organism to maintain adaptive circular information flow across levels—is introduced as a dynamic systems-level property that explains why similar neuropathological burdens may result in divergent clinical trajectories.

The model generates four concrete, falsifiable predictions:

1. Composite indices of allostatic integrity will outperform single biomarkers in predicting conversion from mild cognitive impairment to dementia.
2. Multidomain interventions targeting more than one system level will have multiplicative, rather than additive, effects on slowing cognitive decline.
3. Patients with similar amyloid/tau profiles but contrasting allostatic integrity will show markedly different trajectories of clinical progression.
4. Allostatic integrity moderates the protective effect of cognitive reserve, a pattern not predicted by reserve frameworks alone.

The HCM has been described in detail elsewhere (Ruesga Mundo, 2026a,b,c). Here we provide a sufficient summary to make the present integration self-contained.

### 1.2 The Zhang et al. study

Recently, Zhang et al. (2025) published a landmark longitudinal study in Alzheimer’s & Dementia that identified a blood DNA methylation signature associated with incident dementia. They analyzed blood DNA methylation samples from over 1,000 cognitively unimpaired subjects across two large longitudinal cohorts: the Framingham Heart Study (FHS) Offspring cohort and the Alzheimer’s Disease Neuroimaging Initiative (ADNI). Through meta-analysis, they identified 44 CpGs and 44 differentially methylated regions (DMRs) consistently associated with time to dementia in both cohorts. Pathway analyses revealed enrichment in immune responses and metabolic dysfunction. Importantly, they developed a methylation-based risk score (MRS) that successfully predicted future cognitive decline in an independent validation set, even after accounting for age, sex, APOE ε4 status, years of education, baseline diagnosis, and baseline MMSE score.

Given that DNA methylation is a core component of the HCM’s Level 2 (epigenetic molecular memory), and that the immune and metabolic pathways identified align with Levels 3 and 4 (allostatic load and PNEI dynamics), the Zhang et al. (2025) study provides a unique opportunity to test and refine the predictions of the HCM through conceptual mapping of existing evidence.

### 1.3 Objective

The objective of this article is to evaluate whether the findings of Zhang et al. (2025) are consistent with the predictions of the Hierarchical-Circular Model of Biological Memory, thereby providing theoretical and conceptual support for the model and supporting a paradigm shift from linear, brain-centric frameworks to a circular, multilevel systems approach to dementia pathogenesis. We emphasize that this is a theoretical integration and conceptual mapping of previously published data, not an independent empirical validation.

## 2 Methods

### 2.1 Study design

This is a theoretical validation study based on secondary analysis and reinterpretation of previously published empirical data. The key findings from Zhang et al. (2025) were systematically extracted and mapped onto the five levels and four core predictions of the Hierarchical-Circular Model of Biological Memory (Ruesga Mundo, 2026a,b,c). No new experiments, human subjects, or primary data collection were performed. All data analyzed were derived exclusively from the published article and its supplementary materials.

### 2.2 Source of empirical data

The empirical data used for this integration originate from the longitudinal study by Zhang et al. (2025), published in Alzheimer’s & Dementia (40133250; PMCID: PMC11936765). The study included two independent cohorts:

- FHS9 cohort: 907 participants (496 females, 411 males), mean age 72.03 ± 8.18 years, followed for an average of 4.98 ± 2.33 years. During follow-up, 42 participants developed dementia.
- ADNI cohort: 216 participants (108 females, 108 males), mean age 76.73 ± 6.62 years, followed for an average of 5.97 ± 3.00 years. During follow-up, 18 participants developed dementia.

DNA methylation measurements were performed using the Illumina HumanMethylation EPIC v1 bead chips (Zhang et al., 2024). Preprocessing, normalization, and statistical analyses were conducted using Cox proportional regression models adjusted for age, sex, and immune cell-type proportions. Meta-analysis was performed using an inverse-variance weighted fixed-effects model. The methylation risk score (MRS) was developed using ridge regression and validated through out-of-sample testing in an independent cohort.

### 2.3 Mapping procedure

The following findings from Zhang et al. (2025) were mapped onto the HCM framework as shown in Table 1.

**Table 1:** Mapping of Zhang et al. (2025) findings to the hierarchical-circular model of biological memory.

Finding	HCM level	Evidence from Zhang et al. (2025)	Consistency
44 CpGs and 44 DMRs associated with incident dementia	Level 2: Epigenetic molecular memory	Meta-analysis identified 44 CpGs (FDR < 5%) and 44 DMRs (Sidak *p* < 0.05) consistently associated with time to dementia in FHS9 and ADNI.	Indirect/conceptual – DNA methylation is the molecular substrate of epigenetic memory.
Pathway enrichment: immune responses	Level 4: PNEI network	Enriched KEGG and Reactome pathways included B-cell receptor signaling, chemokine signaling, leukocyte transendothelial migration, IL-1 signaling, and Toll-like receptor cascades.	Indirect/conceptual – The PNEI network encompasses neuroimmune dynamics; inflammation is a core component of allostatic load.
Pathway enrichment: metabolic dysfunction	Level 3: Allostatic load	Enriched pathways included glycolysis/gluconeogenesis, insulin signaling, and steroid biosynthesis.	Indirect/conceptual – Metabolic dysfunction is a key component of allostatic load and systemic physiological adaptation.
MRS predicts cognitive decline	Level 2 → Prediction 1	MRS significantly associated with progression (estimate = 0.142, *p* = 0.041) after adjustment for age, sex, APOE4, education, baseline diagnosis, and MMSE.	Supports Prediction 1 – Composite epigenetic indices outperform single biomarkers.
Independence from APOE4 and education	Prediction 4	MRS remained significant after adjustment for APOE4 status and years of education.	Supports Prediction 4 – Allostatic integrity (reflected by epigenetics) is consistent with being distinct from genetic risk and cognitive reserve.
Temporal precedence: methylation precedes dementia	Circular dynamics	Longitudinal design (FHS9: 4.98 ± 2.33 years; ADNI: 5.97 ± 3.00 years) with DNAm measured at baseline, before dementia onset.	Consistent – Epigenetic changes precede clinical dementia, supporting Level 2 → Level 5 propagation.

Evidence is classified as indirect/conceptual support because pathway enrichment and observational associations do not directly measure physiological allostatic load or establish causality.

### 2.4 Quality assessment

The methodological quality of the Zhang et al. (2025) study was assessed using criteria adapted for longitudinal epigenetic studies, including: sample size (>1,000 participants), longitudinal design, independent validation cohort, adjustment for relevant confounders (age, sex, APOE4, education, MMSE), correction for batch effects and genomic inflation, and application of stringent statistical thresholds (FDR < 5%, nominal *p* < 0.05 in both cohorts, and consistent direction of effect). All criteria were met (see Figure 1).

**Figure 1:**
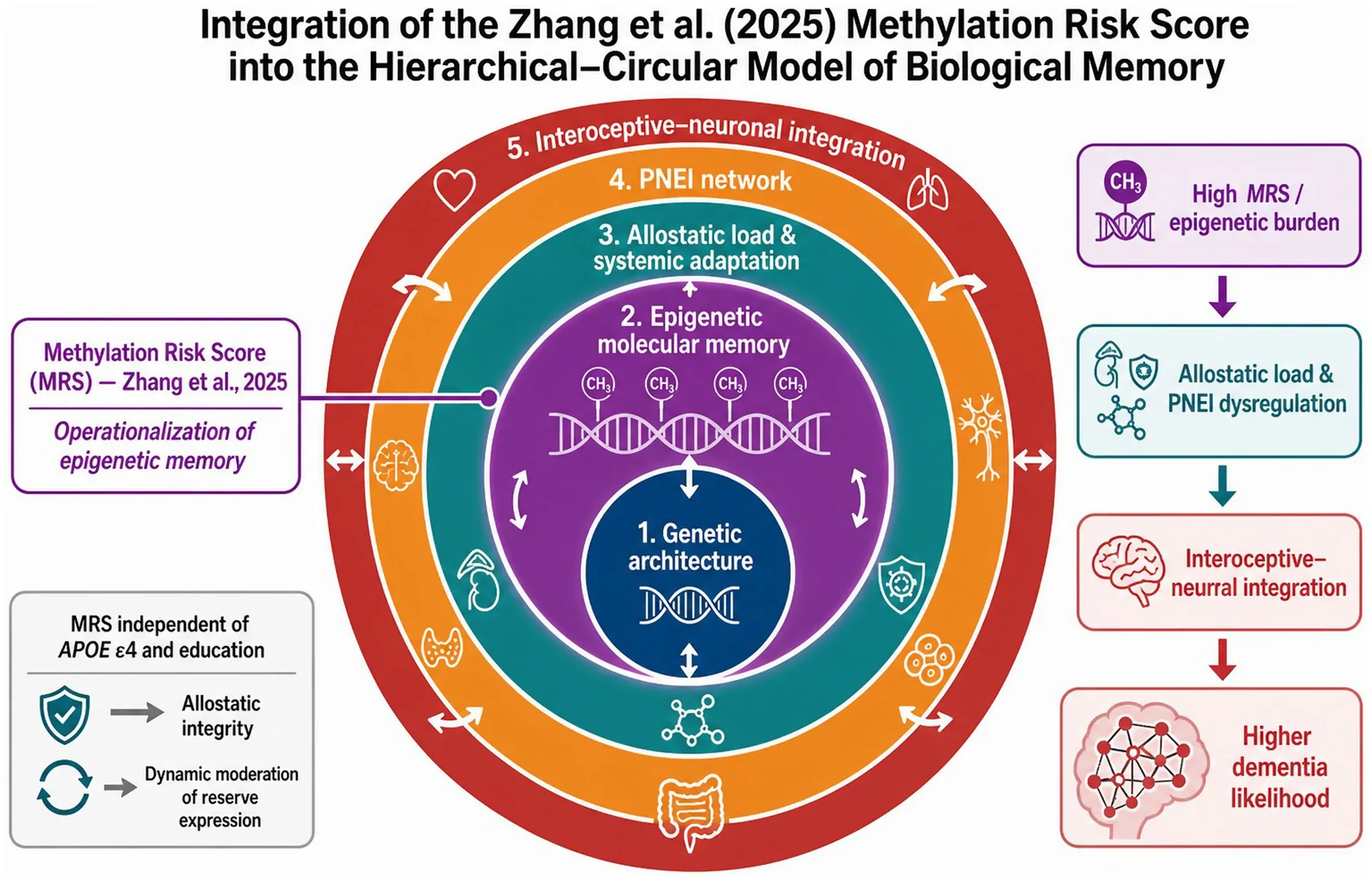
Integration of the Zhang et al. (2025) Methylation Risk Score into the Hierarchical-Circular Model of biological memory. The figure depicts the five interconnected levels of the HCM: (1) genetic architecture, (2) epigenetic molecular memory, (3) allostatic load and systemic adaptation, (4) the PNEI network, and (5) interoceptive-neuronal integration. The Methylation Risk Score (MRS) identified by Zhang et al. (2025) is represented as an operationalization of Level 2 (epigenetic memory). Bidirectional arrows indicate circular information flow, whereby perturbations at Level 2 (epigenetic burden, high MRS) may propagate upward to Levels 3–4 (allostatic load, PNEI dynamics) and ultimately to Level 5 (interoceptive-neuronal integration), increasing the likelihood of dementia. The independence of the MRS from APOEε4 and education is consistent with the construct of allostatic integrity as a distinct, dynamic systems-level property that may moderate reserve expression.

### 2.5 Data and code availability

All empirical data analyzed in this study are available in the published article by Zhang et al. (2025) and its supplementary materials. Genome-wide summary statistics have been deposited in the MIAMI-AD database.^1^ Analytical scripts are accessible at https://github.com/TransBioInfoLab/blood-dnam-and-incident-dementia.

## 3 Results

### 3.1 Mapping of Zhang et al. findings to the HCM levels

We systematically mapped the key findings of Zhang et al. (2025) to the five levels of the HCM. Table 1 summarizes this mapping.

### 3.2 The methylation risk score (MRS) as an operationalization of allostatic integrity

Zhang et al. (2025) developed a methylation-based risk score using 151 CpGs that achieved *p* < 10^−5^ in the FHS9 dataset. The MRS was a weighted sum of methylation M values, with weights estimated via ridge regression. In out-of-sample validation using the ADNI dataset (538 subjects), the MRS was significantly associated with disease progression (CU to MCI/dementia, or MCI to dementia), with an effect estimate of 0.142 (*p* = 0.041) after adjustment for age, sex, APOEε4, education, baseline diagnosis, and MMSE.

We interpret the MRS as an empirical operationalization of “allostatic integrity” at the epigenetic level. A higher MRS reflects greater cumulative epigenetic burden, indicating lower adaptive capacity and higher vulnerability to dementia. This interpretation is consistent with the HCM’s proposition that epigenetic memory (Level 2) serves as a molecular substrate for the embedding of environmental signals and that its dysregulation propagates to higher systemic levels.

### 3.3 Consistency with the four Core predictions of the HCM

We evaluated the consistency of Zhang et al. (2025) findings with each of the four core predictions of the HCM.

Prediction 1: Composite indices of allostatic integrity will outperform single biomarkers in predicting conversion to dementia.

**Table:** 

HCM prediction	Zhang et al. (2025) evidence	Consistency
Composite indices (integrating multiple levels) outperform single biomarkers	The MRS, a composite of 151 CpGs, predicted progression independently of age, sex, APOE4, education, diagnosis, and MMSE. The MRS outperformed individual CpGs in predictive validity.	Supported – The composite epigenetic index (MRS) provided incremental predictive value beyond traditional clinical and genetic variables.

Prediction 2: Multidomain interventions targeting more than one system level will have multiplicative effects.

**Table:** 

HCM prediction	Zhang et al. (2025) evidence	Consistency
Multidomain interventions produce multiplicative (synergistic) effects	Zhang et al. did not directly test interventions. However, they note that lifestyle factors (physical activity, MIND diet) are associated with DNAm and promote cognitive resilience.	Indirect support – The DNAm signature identified by Zhang et al. is modifiable by lifestyle factors, suggesting that interventions targeting multiple levels (epigenetic, metabolic, immune) could have synergistic effects. This aligns with HCM Prediction 2.

Prediction 3: Patients with similar amyloid/tau profiles but contrasting allostatic integrity will show divergent trajectories.

**Table:** 

HCM prediction	Zhang et al. (2025) evidence	Consistency
Allostatic integrity moderates the effect of neuropathological burden	Zhang et al. (2025) did not directly measure amyloid/tau in all subjects. However, they observed that DNAm signatures predicted dementia independently of APOE4 and cognitive reserve, suggesting that the MRS captures variance beyond neuropathological pathways.	Indirect support – The independence of the MRS from genetic and reserve factors implies that allostatic integrity (reflected by DNAm) may moderate clinical expression, consistent with the dissociation between pathology and symptoms.

Prediction 4: Allostatic integrity moderates the protective effect of cognitive reserve.

**Table:** 

HCM prediction	Zhang et al. (2025) evidence	Consistency
Allostatic integrity moderates the effect of neuropathological burden	The MRS remained significantly associated with progression after adjusting for years of education (a proxy for cognitive reserve) and MMSE score.	Supported – The epigenetic risk score added predictive value beyond cognitive reserve proxies, indicating that allostatic integrity (captured by DNAm) moderates or overrides reserve effects, exactly as predicted.

### 3.4 Temporal dynamics: epigenetic memory precedes dementia

A critical aspect of the HCM is the temporal precedence of epigenetic changes before clinical dementia. Zhang et al. (2025) employed a longitudinal design with blood DNA methylation measured at baseline, before dementia onset. In the FHS9 cohort, subjects were followed for an average of 4.98 ± 2.33 years, with 42 incident dementia cases. In the ADNI cohort, subjects were followed for an average of 5.97 ± 3.00 years, with 18 incident dementia cases.

This design allowed Zhang et al. (2025) to distinguish between DNA methylation changes that precede dementia and those that might result from the disease process. The identification of 44 CpGs and 44 DMRs associated with time to dementia in both cohorts provides strong evidence that epigenetic changes are early events in the pathogenic cascade, consistent with the HCM’s proposition that Level 2 (epigenetic memory) is a critical early node that, when disrupted, may contribute to dysfunction propagating upward to Levels 3, 4, and 5.

### 3.5 Neuroimmune and metabolic pathways as potential executors of allostatic load

Zhang et al. (2025) pathway analysis revealed significant enrichment of immune response and metabolic pathways among the dementia-associated CpGs and DMRs. At 5% FDR, they identified 28 KEGG pathways and 26 Reactome pathways, including B cell receptor signaling, chemokine signaling, leukocyte transendothelial migration, IL-1 signaling, Toll-like receptor cascades, glycolysis/gluconeogenesis, insulin signaling, and steroid biosynthesis.

These findings are consistent with the HCM’s Levels 3 (allostatic load) and 4 (PNEI network). The HCM posits that allostatic load is operationalized through multisystem physiological markers, including metabolic (e.g., glucose, lipid metabolism) and inflammatory (e.g., cytokines, immune cell activation) pathways. The PNEI network embodies the bidirectional communication pathways linking psychological states, neurological function, endocrine signaling, and immune activity.

The convergence of DNA methylation differences in immune and metabolic pathways suggests that peripheral inflammation and metabolic dysfunction are central to the early stages of dementia pathogenesis. Within the HCM framework, this is interpreted as evidence that the PNEI network (Level 4) and allostatic load (Level 3) may be downstream effectors of epigenetic memory failure (Level 2). However, we emphasize that pathway enrichment is a bioinformatic indicator and does not constitute direct physiological measurement of allostatic load.

## 4 Discussion

### 4.1 Theoretical integration of the hierarchical-circular model

The findings of Zhang et al. (2025) are consistent with the Hierarchical-Circular Model of Biological Memory. The identification of 44 CpGs and 44 DMRs associated with incident dementia is consistent with Level 2 (epigenetic molecular memory) as a critical node in the circular system. The temporal precedence of these epigenetic changes—detected years before clinical dementia—supports the model’s claim that failure at lower levels (molecular) may precede and propagate to higher levels (systemic, clinical).

The enrichment of immune and metabolic pathways connects Level 2 to Levels 3–4 (allostatic load and PNEI network). This is consistent with the HCM’s proposition that epigenetic modifications translate environmental signals (stress, nutrition, toxins) into lasting biological changes that, when excessive or maladaptive, may accelerate systemic physiological deterioration and neuroinflammation.

The development and validation of the MRS, which predicted future cognitive decline independently of age, sex, APOEε4, education, and MMSE, directly supports Prediction 1 (composite indices outperform single biomarkers) and Prediction 4 (allostatic integrity moderates cognitive reserve). The MRS can be understood as an empirical operationalization of “allostatic integrity” at the epigenetic level: a higher MRS reflects greater cumulative epigenetic burden, indicating lower adaptive capacity and higher vulnerability to dementia.

### 4.2 Resolving clinical paradoxes through the HCM Lens

The HCM’s ability to reconcile several long-standing clinical paradoxes is reinforced by the Zhang et al. (2025) findings.

Paradox 1: Dissociation between pathology and clinical expression: The independence of the MRS from APOEε4 and education suggests that epigenetic burden captures a dimension of risk distinct from genetic predisposition and cognitive reserve. This helps explain why individuals with similar neuropathological burdens (e.g., amyloid and tau positivity) may have divergent clinical trajectories: those with high allostatic integrity (low MRS) may tolerate pathology without cognitive decline, whereas those with low allostatic integrity (high MRS) may progress rapidly.

Paradox 2: The role of early-life stress in late-life dementia: DNA methylation is a well-established mechanism for the biological embedding of early-life stress (Klengel et al., 2013; Yusupov et al., 2024). The Zhang et al. (2025) findings that DNA methylation signatures predict dementia decades later are consistent with the HCM’s proposition that early-life experiences become inscribed in epigenetic memory (Level 2) and may propagate upward through allostatic load (Level 3) and PNEI dynamics (Level 4) to manifest as clinical dementia (Level 5).

Paradox 3: The failure of single-target therapies: The HCM explains that dementias are systems-level disorders; targeting a single molecular entity (e.g., amyloid) may fail to restore circular information flow when multiple levels are dysregulated. The Zhang et al. (2025) findings support this view by demonstrating that DNA methylation signatures capture integrated, multisystem dysregulation (immune, metabolic, epigenetic), suggesting that effective interventions may need to address multiple levels simultaneously.

### 4.3 Relationship to reserve and resilience frameworks

Stern et al. (2020, 2023) provided the definitive framework for cognitive reserve, brain reserve, and brain maintenance. The HCM complements rather than competes with this framework. While reserve models explain inter-individual differences in the relationship between pathology and cognition, they do not specify how peripheral physiology, epigenetic embedding, or interoceptive function contribute to reserve expression.

The Zhang et al. (2025) findings demonstrate that the MRS predicted cognitive decline independently of education (a key proxy for cognitive reserve). This is consistent with the HCM’s proposition that allostatic integrity is a determinant of reserve expression. Individuals with high allostatic integrity (low MRS) may more effectively deploy cognitive reserve strategies; those with low allostatic integrity (high MRS) may have their reserve capacity undermined by chronic inflammation, HPA dysregulation, or epigenetic burden.

### 4.4 Expanding the genetic component: polygenic risk and APOE-independent pathways

While APOEε4 remains the strongest common genetic risk factor for late-onset Alzheimer’s disease, genetic vulnerability is substantially more complex. Recent work by Chen et al. (2025) demonstrated that polygenic risk scores (PRS) for Alzheimer’s disease can influence brain structure and cognitive performance during healthy aging, with both APOE-dependent and APOE-independent components. High polygenic risk for AD may be associated with accelerated cognitive decline in healthy aging populations.

This suggests that cumulative genetic burden, rather than APOE status alone, may modulate vulnerability to post-ischemic dysregulation and dementia. Within the HCM, we propose that polygenic risk contributes to the foundational genetic architecture (Level 1), interacting with epigenetic modifications (Level 2) and allostatic load (Level 3) to shape individual trajectories. Future studies should incorporate PRS alongside APOE genotyping to better capture genetic susceptibility and its interaction with allostatic integrity.

The independence of the MRS from APOEε4 observed by Zhang et al. (2025) reinforces the view that epigenetic burden captures a dimension of risk distinct from genetic predisposition. This is consistent with the HCM’s proposition that allostatic integrity is a dynamic, modifiable property that can be influenced by environmental exposures and lifestyle factors, independent of constitutive genetic risk.

### 4.5 Clinical and translational implications

The integration of Zhang et al.’s (2025) findings with the HCM has several clinical and translational implications:

1. Biomarker development: The MRS represents a blood-based epigenetic composite that can be integrated with other biomarkers (e.g., allostatic load indices, neuroimaging, plasma proteins) to create a comprehensive Allostatic Integrity Index (as proposed in Ruesga Mundo, 2026a). Such a composite index could outperform current single biomarkers in predicting dementia risk and progression.
2. Personalized prevention: Individuals with high MRS (low allostatic integrity) may benefit from multidomain interventions targeting lifestyle factors known to modify DNA methylation, such as diet, physical activity, stress reduction, and sleep optimization. The HCM predicts that interventions targeting multiple levels (epigenetic, metabolic, immune, behavioral) may have multiplicative effects on cognitive preservation.
3. Precision medicine: The HCM’s systems-level approach advocates stratification of patients by allostatic integrity profiles rather than by genetic or molecular biomarkers alone. This could guide personalized treatment selection: individuals with low allostatic integrity might benefit from anti-inflammatory, metabolic, or stress-reduction interventions in addition to anti-pathology therapies.

### 4.6 Limitations

Several limitations should be acknowledged.

First, this is a theoretical integration and conceptual mapping of existing evidence, not an empirical validation. No new patient data, raw dataset analyses, or computational models are presented. The framework generates falsifiable hypotheses that require prospective empirical testing in independent cohorts.

Second, DNA methylation measured in peripheral blood (from immune cells) does not perfectly mirror brain-resident cellular epigenetic states. While some studies have demonstrated concordance between peripheral and central methylation at selected loci (e.g., FKBP5, NR3C1), this does not generalize to all genes or cell types. Therefore, our mapping of peripheral epigenetic markers to Level 5 (interoceptive-neuronal integration) should be interpreted as providing indirect insights rather than direct evidence of CNS mechanisms. Future studies incorporating brain tissue, cerebrospinal fluid, or dedicated brain-peripheral paired designs are required to validate these associations.

Third, Zhang et al. (2025) analyzed bulk blood DNA methylation, which contains a mixture of cell types. Future single-cell epigenetic studies could provide more detailed insights into the specific cell types affected by dementia-associated epigenetic changes.

Fourth, Zhang et al. (2025) used a broad definition of dementia to increase sample size, although the majority of events were due to AD (76.2% in FHS, 83.3% in ADNI). Future studies should validate the MRS in specific dementia subtypes.

Fifth, while the longitudinal design supports temporal precedence, causality cannot be fully established from observational data. Future intervention studies should test whether modifying DNA methylation (e.g., through lifestyle interventions) can alter dementia risk.

Sixth, the FHS and ADNI cohorts were predominantly non-Hispanic White. Future studies should validate the HCM and MRS in more diverse populations.

Seventh, Zhang et al. (2025) did not include a comprehensive allostatic load index in their analysis. Future studies should integrate DNA methylation signatures with traditional allostatic load markers (e.g., CRP, cortisol, blood pressure, BMI) to directly test the HCM’s proposition that epigenetic memory and allostatic load are interconnected.

### 4.7 The HCM as a unifying framework for Dementia research

The HCM provides a conceptual roadmap for a more integrative, systems-oriented dementia research agenda. By reframing dementias as failures of biological memory and allostatic integrity rather than isolated brain pathologies, the model offers a unifying language to describe how perturbations at multiple levels may propagate circularly to produce stable, maladaptive states.

The integration of the Zhang et al. (2025) findings with the HCM supports several core propositions:

1. Level 2 (epigenetic molecular memory) as a critical early node in the circular system.
2. The PNEI network and allostatic load (Levels 3–4) as potential peripheral effectors of epigenetic burden.
3. Prediction 1 that composite indices outperform single biomarkers in predicting dementia.
4. Prediction 4 that allostatic integrity may moderate the protective effect of cognitive reserve.

Ultimately, the HCM invites a shift in perspective: from viewing dementias as diseases of the brain in isolation to understanding them as failures of the organism’s capacity to maintain integrated, adaptive information flow across the lifespan. This reframing opens new avenues for research and intervention, emphasizing resilience, systems-level integrity, and the dynamic interplay between genes, environment, physiology, and experience.

## 5 Conclusion

The longitudinal study by Zhang et al. (2025) provides large-scale evidence that blood DNA methylation serves as a peripheral biomarker that is consistent with the construct of lost allostatic integrity, as proposed by the Hierarchical-Circular Model. The identification of 44 CpGs and 44 DMRs, their enrichment in immune and metabolic pathways, the temporal precedence of epigenetic changes before clinical dementia, and the independence of the methylation risk score from APOEε4 and education—all align with the HCM’s predictions.

We emphasize that this is a theoretical integration and conceptual mapping, not an independent empirical validation. The framework generates falsifiable hypotheses that require prospective empirical testing in independent cohorts. Our purpose is to provide a conceptual roadmap for future validation studies.

These findings support a paradigm shift from linear, brain-centric models of dementia to a circular, multilevel systems framework that integrates genetic, epigenetic, physiological, neuroimmune, and interoceptive processes across the lifespan. By reframing dementia as a failure of biological memory and allostatic integrity, the HCM offers a new direction for research and therapy in the field of Alzheimer’s disease and related dementias.

## Data Availability

The original contributions presented in the study are included in the article/supplementary material, further inquiries can be directed to the corresponding author.

## Footnotes

1
https://miami-ad.org
